# Case Report: Genetic and Clinical Features of Maternal Uniparental Isodisomy-Induced Thiamine-Responsive Megaloblastic Anemia Syndrome

**DOI:** 10.3389/fped.2021.630329

**Published:** 2021-03-19

**Authors:** Pengjiang Kang, Weihua Zhang, Jinquan Wen, Jiming Zhang, Fei Li, Wuxia Sun

**Affiliations:** Caihong Hospital, Xianyang, China

**Keywords:** thiamine, diabetes, megaloblastic anemia syndrome, SLC19A2, case report

## Abstract

**Background:** Thiamine-responsive megaloblastic anemia syndrome (TRMA) is a rare autosomal recessive hereditary disease due to mutations in *SLC19A2*. Some cases show familial inheritance.

**Case report:** A female patient (from a gravida 1, para 1 mother) of 3.5 years of age was admitted to the Pediatric Hematology Department of Xianyang Caihong Hospital in June 2019. The patient had severe anemia, acupoint-size bleeding spots, and a few ecchymoses all over her body, as well as astigmatism and hyperopia. Hearing was normal. The patient had diabetes. Bone marrow biopsy suggested a myelodysplastic syndrome. The patient had a c.515G>A (p.G172D) homozygous mutation of *SLC19A2* (NM_006996), indicating TRMA. Genetic testing revealed that the two alleles were inherited from her mother alone due to maternal uniparental isodisomy (UPD). The patient was treated with thiamine and a subcutaneous injection of insulin. The patient recovered well and was discharged. She continued thiamine and insulin at the same dose and was followed once a month. The last follow-up on September 15, 2020, showed no anemia or bleeding. She had a sound hearing and normal blood routine and fasting glucose levels. Hyperopia and astigmatism did not improve.

**Conclusion:** The patient had TRMA induced by the c.515G>A (p.G172D) homozygous mutation of *SLC19A2* inherited through maternal UPD. The genetic diagnosis of TRMA is of significance for guiding clinical treatment. Early treatment with exogenous thiamine can improve some of the clinical features of TRMA.

## Introduction

Thiamine-responsive megaloblastic anemia syndrome (TRMA), first reported by Porter et al. in 1969 (1), is a rare autosomal recessive hereditary disease with an extremely low incidence. Only about 80 cases have been reported worldwide so far (2). TRMA is characterized by megaloblastic anemia, diabetes, and sensorineural deafness and is also associated with congenital heart disease, arrhythmia, cardiomyopathy, retinal detachment, optic atrophy, and stroke. The average time to diagnosis is up to 8 years (3). The early diagnosis of TRMA is based on family history, apoptosis in fibroblast during culture without thiamine, and thiamine therapy's effectiveness (4). TRMA is induced by mutations in *SLC19A2* (NM_006996.2) that encodes the high-affinity thiamine transporter-1 (THTR-1) (5, 6). So far, >60 mutation reports have been reported (Table 1) (7). In this report, the genetic and clinical features of the first TRMA patient worldwide with the c.515G>A (p.G172D) *SLC19A2* homozygous mutation inherited through maternal uniparental isodisomy (UPD) are reported.

**Table 1:** Comparsion of the data of patients with TRMA.

**Number**	**Publish time**	**Author**	**Country**	**Gender**	**Age at diagnosis**	**Deafness**	**Diabetes**	**Anemia**	**Other symptoms**	***SLC19A2* mutations**
1	1969	Rogers	USA	Female	11 years	+	+	+	Aminoaciduria	
2	2008	Ediz	Turkish	Female	2 years	+	+	+		697 C_T
3	2011	Zehra	Turkish	Male	8 years	+	+	+	Atrial standstill	1147del GT
4	2013	Mozzillo	UK	Female	20 month	+	+	+	Optic atrophy	c.242 ins A
5	2013	Mozzillo	UK	Female	27 month	+	+	+	Ocular abnomality	c.1370 del T
6	2016	Mustafa	Turkish	Male	5 years	+	+	+	Ocular abnomality	697C>T
7	2016	Mustafa	Turkish	Female	7 month	+		+		1105delITT
8	2016	Mustafa	Turkish	Female	18 month	+		+	Optic atrophy	1105delITT
9	2016	Mustafa	Turkish	Female	4 month	+	+	+	Optic atrophy	566_567delIGCinsTCT
10	2016	Mustafa	Turkish	Male	16 month	+	+	+		566_567delIGCinsTCT
11	2017	QingCheng	China	Male	6 years	+	+	+		c.903 del G
12	2018	Xiaoying	China	Male	4 months		+	+	Visual impairment	c.1409 ins T
13	2018	Isik	Turkish	Male	5 years		+	+	Thrombocytopenia	1265 T>C
14	2018	Samaher	Saudi Arabia	Female	4 months	+		+		c.1366_1 G>C
15	2020	JinquanWen	China	Female	3.5 years		+	+		c.515G>A (P. G172D)

## Case Presentation

**Figure 1:**
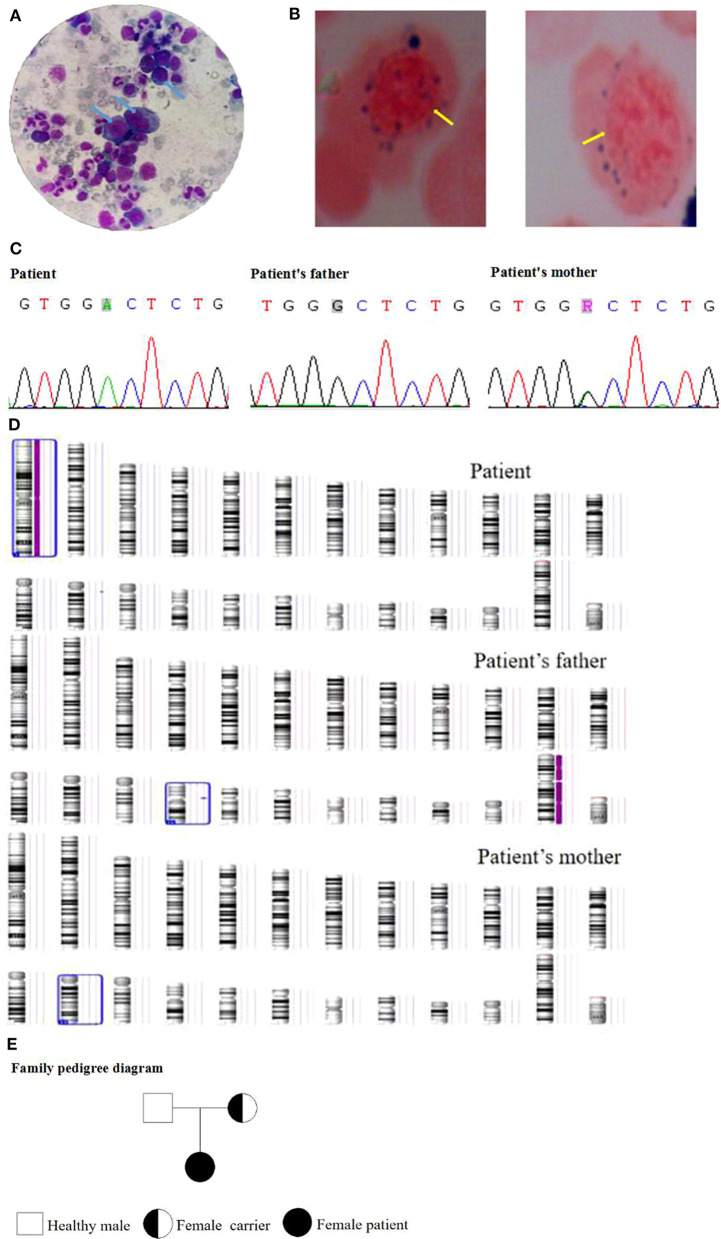
**(A)** Bone marrow examination of the patient. **(B)** Erythrocytic examination of the patient. **(C)** Gene mutation of the family. **(D)** Gene chip detection of the family. **(E)** The family pedigree.

A female patient (from a G1P1 mother) of 3.5 years of age was admitted to the Pediatric Hematology Department of Xianyang Caihong Hospital in June 2019. Body temperature was 36.7°C, and body weight was 15 kg. The patient had severe anemia, acupoint-size bleeding spots, and a few ecchymoses all over her body, as well as astigmatism and hyperopia. Hearing was normal. There were no palpable superficial lymph nodes. Heart and lung auscultation showed no abnormalities. No abnormalities were observed in the limbs. Growth and development appeared normal for age. Both parents were in good health. It was a non-consanguineous marriage. There was no familial history of diabetes and hereditary anemia. Laboratory routine blood test showed white blood cells (WBC) at 5.48 × 10^9^/L, neutrophils (N) at 26.64%, absolute neutrophil count (ANC) at 1.46 × 10^9^/L, lymphocytes (L) at 61.5%, red blood cells (RBC) at 1.66 × 10^12^/L, hemoglobin (Hb) at 51 g/L, mean corpuscular volume (MCV) at 98 fl, platelets (PLT) at 8 × 10^9^/L, and Ret at 0.022. Biochemical examination showed fasting blood glucose at 13.0 mmol/L (3.9–6.1 mmol/L), fasting insulin at 1.02 mol/L (3–25 mol/L), and HbA1c at 7.8% (4.0–6.5%), indicating diabetes. Serum ferritin, folic acid, and vitamin B12 levels were normal. Bone marrow examination revealed hyperplasia, with high erythrocyte proliferation, accounting for 48.5%, with 24.5% of megaloblasts (Figure 1A). There were vacuoles in the cytoplasm of some primitive erythrocytes. No pathological hematopoiesis was observed in granulocytes and megakaryocytes. Erythrocytic examination showed iron particles in the extracellular area, as well as ringed sideroblasts (13%) (Figure 1B). Bone marrow biopsy suggested myelodysplastic syndrome (MDS). The karyotype analysis showed 46, XX. The chromosome mutagenicity testing showed that the patient had DNA damage in peripheral lymphocytes. The comet assay showed that the comet cell percentage was 21% and that there was no apoptosis. The mitochondrial gene testing was not consistent with the patient's phenotype or suspected pathogenic mutation. Peripheral blood samples of the patient and her parents were collected for genomic DNA analysis. The patient had a c.515G>A (p.G 172D) homozygous mutation in *SLC19A2* (NM_006996), indicating TRMA. Exon 2 of *SLC19A2* was amplified and sequenced (*SLC19A2*-2F: CGG GAG GCT CCT CCA TAT TT; *SLC19A2*-2R: TTG CCA CAG ACT ACC TCC GT), and the results showed that the mother was a carrier of the c.515G>A (p.G 172D) heterozygous mutation and that the father was wild type (Figure 1C). There was a homozygosis zone of 248.31 Mb in the region of homozygosity [ROH (1p36.33q44)] [i.e., region arr (hg19) 1p36.33q44 (888,658–249,198,16)hmz], while chromosome 1 was of the normal copy number (Figure 1D). No database-recorded and disease-related CNV changes were observed in her father and mother according to the gene chip detection results, indicating that the patient's homozygosis region was inherited from her mother alone and induced by maternal UPD. The patient was treated with thiamine (10 mg/time, 3 times/day, Linfen Baozhu Pharmaceutical) and subcutaneous injection of insulin of 6 U (i.h, 3 times/day before each meal). Blood glucose levels were maintained at 4.1–10 mmol/L. After 10 days of treatment, blood routine showed that Hb had increased from 54 to 112 g/L, reticulocytes from 2.2 to 14%, and PLT from 8 × 10^9^/L to 217 × 10^9^/L. The insulin dose was adjusted to 3 U (i.h, 3 times per day, before each meal). Thiamine was continued (10 mg/ day, 3 times per day). Fasting glucose levels were maintained at 3.5–7.2 mmol/L.

The patient recovered well and was discharged. She continued to receive treatment and follow-up once a month. The last follow-up on September 15, 2020, showed no anemia or bleeding. She had a sound hearing and normal blood routine and fasting glucose. Hyperopia and astigmatism did not improve significantly. The family pedigree is shown in Figure 1E.

## Discussion

This patient's clinical manifestations were anemia, hemorrhage, diabetes, hyperopia, astigmatism, megaloblastic anemia with ringed sideroblasts, and severe thrombocytopenia, which are the main symptoms of TRMA. Molecular genetics revealed the c.515G>A(p.G 172D) homozygous mutation in *SLC19A2*. Sanger sequencing and gene chip proved that the disease was caused by maternal UPD. Thiamine was effective. Thus, the patient was diagnosed with TRMA. Four TRMA patients have been reported to have anemia and diabetes (8–10). Among them, two were siblings (8), whose gene detections both showed C.1409insT homozygous mutation of Gene *SLC19A2*. The other two were unrelated by blood, one of which had paternal c.484 C>T and maternal c.1001 G>A compound heterozygous mutation of *SLC19A2* (9), and the other was complicated with eye diseases (8). All 4 patients presented mild/moderate anemia. Borgna-Pignatti et al. (11) and Labay et al. (12) reported, respectively, 2 patients (who were cousins) in a family with the c.515G>A (p.G 172D) mutation in *SLC19A2*. Clinical manifestations were anemia (mild-to-moderate), diabetes, deafness, growth and development retardation, eye diseases, and megaloblasts and ringed sideroblasts in the bone marrow. After 2 weeks of thiamine treatment, their hemocytes returned to normal, and their insulin dosage was reduced (11, 12). The patient reported here presented the clinical manifestations of severe anemia and thrombocytopenia, but no deafness, growth and development retardation, or eye diseases. Therefore, this patient was not completely identical to the other patients with a genetic variation at this site. It could be due to racial differences, but the exact reason is unknown. The gene chip assay showed that the patient had a homozygosis zone of 248.31 Mb in ROH (1p36.33q44), inherited from her mother. It was speculated that this case was induced by maternal UPD involved c.515G>A (p.G 172D) homozygous mutation of *SLC19A2*. The combined application of high-throughput sequencing, first-generation sequencing, and gene chip detection enabled the complete and accurate diagnosis of TRMA and the identification of its pathogenesis. Cytological function verification testing showed that the c.515G>A (p.G 172D) mutation of *SLC19A2* might cause the misfolding of the THTR-1 protein that cannot then be completely glycosylated and locate to the target cell membrane, failing to transport thiamine and impaired thiamine absorption (13).

Therefore, it was speculated that the DNA damage in the peripheral lymphocytes in this patient might be caused by intracellular thiamine deficiency due to the mutation of *SLC19A2* in lymphocytes. Several UPD cases were reported regarding ROH (1p36.33q44), all of which were accompanied by clinical chromosome 1 abnormalities. In dense osteogenesis imperfecta, the primary gene involved is CTSK, and the patients mainly present anterior fontanelle closure delay, multiple fractures, short stature, osteosclerosis, and acroosteolysis (14, 15). A case with speckled dyschondroplasia had changes in the GNPAT gene and mainly presented proximal long bone shortening, cataract, abnormal facial appearance, development retardation, and cerebellar atrophy (16). According to reports of cases with the Hutchinson-Gilford progeria syndrome, the primarily involved gene is LMNA, and the patients mainly present short stature, joint degeneration, and atherosis (17, 18). In patients with Zellweger syndrome, the primarily involved gene is PEX1, and the patients mainly present hypotonia, hepatic enlargement, abnormal facial bone development, epilepsy, and craniofacial abnormalities (19). Patriarchal sex single diploid involving A III genes results in glycogen III glycogen storage disease and severe dysplasia (20). The case reported here, induced by UPD in ROH (1p36.33q44), developed none of the clinical manifestations described above. Unlike the other cases, the major gene involved in this case was *SLC19A2*, which is the first UDP report in *SLC19A2*, causing TRMA. Unlike typical TRMA, the patient reported here had severe thrombocytopenia, no deafness, growth retardation, no heart disease, and no neurological disease. The patient had no deafness, which might be because she was young, or it might be a new feature of UPD-induced c.515G>A(p.G172D) mutation of *SLC19A2*. After 10 days of oral thiamine, the Hb and PLT levels returned to normal, the reticulocytes increased, and the requirement for insulin for blood glucose control could be decreased. The treatment was effective.

In conclusion, the patient had TRMA induced by maternal-UPD-involved c.515G>A(p.G172D) homozygous mutation of *SLC19A2*. The genetic diagnosis of TRMA is of significance for guiding clinical treatment. Early treatment with exogenous thiamine can improve some of the clinical features of TRMA.

## Data Availability Statement

The original contributions presented in the study are included in the article/supplementary material, further inquiries can be directed to the corresponding author/s.

## Ethics Statement

The studies involving human participants were reviewed and approved by the Medical Ethics Committee of Xianyang Caihong Hospital. Written informed consent from the participants' legal guardian/next of kin was not required to participate in this study in accordance with the national legislation and the institutional requirements.

## Author Contributions

PK and JW contributed to conception, design of the study, and polished the manuscript. WZ organized the database. FL and WS followed up the patient management. PK wrote the first draft of the manuscript. All authors contributed to manuscript revision, read, and approved the submitted version.

## Conflict of Interest

The authors declare that the research was conducted in the absence of any commercial or financial relationships that could be construed as a potential conflict of interest.
